# Selective Isolation of Trypsin Inhibitor and Lectin from Soybean Whey by Chitosan/Tripolyphosphate/Genipin Co-Crosslinked Beads

**DOI:** 10.3390/ijms15069979

**Published:** 2014-06-04

**Authors:** Yu-Lung Chang, Tristan C. Liu, Min-Lang Tsai

**Affiliations:** Department of Food Science, National Taiwan Ocean University, 2 Pei-Ning Road, Keelung 20224, Taiwan; E-Mails: kevinchang813@gmail.com (Y.-L.C.); tristan.c.liu@gmail.com (T.C.L.)

**Keywords:** chitosan/tripolyphosphate/genipin bead, selective isolation, trypsin inhibitor, lectin, soybean whey

## Abstract

Selective isolation of Kunitz trypsin inhibitor (KTI) and lectin from soybean whey solutions by different types of chitosan beads was investigated. The chitosan beads were co-crosslinked with tripolyphosphate/genipin in solutions at pH 5, 7 or 9 (CB5, CB7, CB9). The maximum adsorption ratios of chitosan beads to KTI and lectin were observed at pH 4.4 and 5.4, respectively; highly selective separation was also demonstrated at these pHs. The adsorption ratios increased with temperature, rising between 5 and 25 °C. CB9 produced the best adsorption ratio, followed by CB7 then CB5. The critical interaction governing absorption of chitosan beads to KTI and lectin could be hydrogen bonding. At pH 9, KTI and lectin desorbed efficiently from CB7 with desorption ratios of 80.9% and 81.4%, respectively. The desorption was most likely caused predominantly by electrostatic repulsion. KTI and lectin can effectively be selectively isolated from soybean whey using this novel separation technique.

## 1. Introduction

Soy is one of the principal foods consumed in most Asian countries. Currently, soy consumption is increasing in western countries since it is a good source of vegetable protein, contains all essential amino acids required for human nutrition, and has low fat content [1]. Soybean whey, a by-product from the preparation of soybean food stuffs such as tofu and soy protein isolates, contains 0.43% (*w*/*v*) protein and also includes several specific protein compounds like trypsin inhibitor, lectin, lipoxygenase, urease and β-amylase [2,3]. In order to increase the added value of soy by-products and reduce process waste, it is urgently necessary to develop an effective approach to recover functional proteins from soy whey.

The isoelectric point (pI) of soybean trypsin inhibitor is pH 4.5. Soybean trypsin inhibitor can be divided into Kunitz trypsin inhibitor (KTI) and Bowman-Birk inhibitor, and their molecular weights are 20 kDa [4] and 8 kDa [3], respectively. The KTI has two disulfide bridges and is very stable between pH 3 to 10, and has a relatively high thermal stability in low moisture conditions [5]. The trypsin inhibitor showed effects on carcinogenesis in a variety of *in vivo* and *in vitro* systems. This may be due to the dietary trypsin inhibitor inducing synthesis and distribution of endogenous trypsin inhibitor (acute-phase reactants), which have widespread effects on cell growth and behavior. Topical administration of trypsin inhibitor also demonstrated prominent anti-inflammatory effects [6].

Soybean agglutinin (lectin) is a tetramer (120 kDa) with a pI of 5.81. It is composed of identical subunits (*M*_W_ 30 kDa each) lacking disulfide bridges and having two *N*-acetyl-d-galactosamine binding sites [3,7]. Lectins are found in every kingdom of life. They have been utilized in many applications of biological and biomedical research due to their high affinity and specificity for glycoconjugates, including cell-surface glycoconjugates, blood typing, host-pathogenic interaction, cancer metastasis, cell-cell communication, embryogenesis, mitogenic stimulation and tissue development [8].

Several purification approaches focused on trypsin inhibitors and lectins have been proposed; they include electrophoresis [9], affinity chromatography [10,11,12,13], ion exchange chromatography [14], liquid-liquid extraction using reversed micelles [15], aqueous two-phase systems [16], integration of affinity precipitation with aqueous two-phase affinity extraction [17] and superparamagnetic microbeads method [18,19]. An economical, quick and effective technique to purify trypsin inhibitors and lectins would benefit not only biological research, but also industrial applications.

Chitosan is a polysaccharide with high molecular weight linked by β-1,4 glucoside and composed of *N*-acetyl-glucosamine and glucosamine. It is often used as an adsorbent for protein, dye, metal, lipid *etc.* [20,21,22,23,24,25]. The amino, hydroxyl, and *N*-acetyl reactive groups on chitosan structure could interact with adsorbates by electrostatic interaction, hydrogen bonding and hydrophobic interaction. Tripolyphosphate (TPP) and genipin are co-crosslinkers. The TPP can ionize and interact with adsorbates via electrostatic interaction. Genipin can interact with adsorbates through hydrogen bonding due to the presence of hydroxyl groups in the structure of genipin.

Selective adsorptions of chitosan have been extensively studied. Vold *et al.* [24] reported that chitosan showed a strong selectivity towards molybdate polyoxyanions with selectivity coefficients around 100, as well as a strong selectivity towards Cu^2+^ compared to Zn^2+^, Cd^2+^ and Ni^2+^, with selectivity coefficients from 10 to 1000. Casal *et al.* [20] used chitosan to selectively remove β-lactoglobulin (β-LG) from cheese whey based on electrostatic interactions between whey proteins and chitosan. At pH 6.2, β-LG could be completely removed by chitosan (1.9 to 3.0 mg/mL), whereas at least 80% of the rest of whey proteins remained in the solution. Furthermore, Montilla *et al.* [22] recovered 90% β-LG with a protein purity of 95% by adjusting the pH of the β-LG-chitosan complex solution to 9. Sepehran *et al.* [23] reported that raw and formaldehyde modified chitosan can selectively adsorb Cu^2+^ and Ni^2+^ ions from their mixture solution. The adsorption selectivity of chitosan can be enhanced by chemical treatment and regulating pH and contact time. Among these factors, chemical modification of chitosan was the most effective factor for the ratio of Ni^2+^ and Cu^2+^ removal efficiency. Feng *et al.* [21] reported that both ovalbumin and lysozyme could be effectively adsorbed on the chitosan/carboxymethylchitosan (CMCS) membrane. The pH, the initial protein concentration and the CMCS content in the membrane affected the adsorption capacities of the membrane. Due to the amphoteric property of protein and membrane, both ovalbumin and lysozyme could be selectively separated from the mixture solution by adjusting the pH of the feed and desorption solutions.

In our previous study, selective adsorption of phytic acid was achieved via electrostatic interaction by chitosan/tripolyphosphate/genipin co-crosslinked beads (CB7) in pH 2 soybean whey solution at 25 °C. The highest adsorption ratio for phytic acid was 30.23%, but KTI and lectin were virtually not adsorbed. Additionally, the highest desorption ratio of phytic acid from the beads was 93.98% in pH 9 solution [25].

Selective isolation of protein has more challenges than that of smaller molecules such as phytic acid, dyes and metals because proteins have diverse structures, and complex interactions between adsorbent and adsorbate occur. Furthermore, development of operative conditions will be required to scale-up the process for practical application. In this study, adsorption and desorption of KTI and lectin by different types of chitosan/TPP/genipin co-crosslinked beads (CBs) from soybean whey solutions at different pHs and temperatures were explored. Furthermore, the feasibility of selective isolation for KTI and lectin is assessed.

## 2. Results and Discussion

### 2.1. Effect of pH

**Figure 1:**
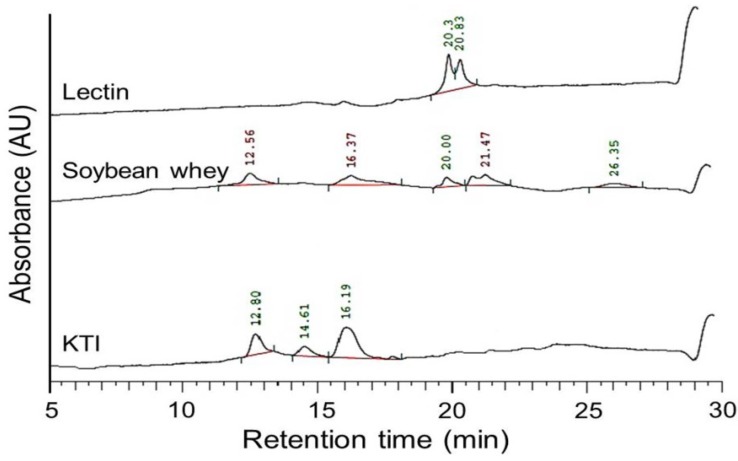
The HPLC elution patterns of Kunitz trypsin inhibitor (KTI), lectin and soybean whey.

Figure 1 shows the HPLC elution patterns of KTI, lectin and soybean whey. The peaks showing retention times of soybean whey were nearly the same as those of the KTI and lectin standards. These results indicate that the major proteins in soybean whey were KTI and lectin. 

**Figure 2:**
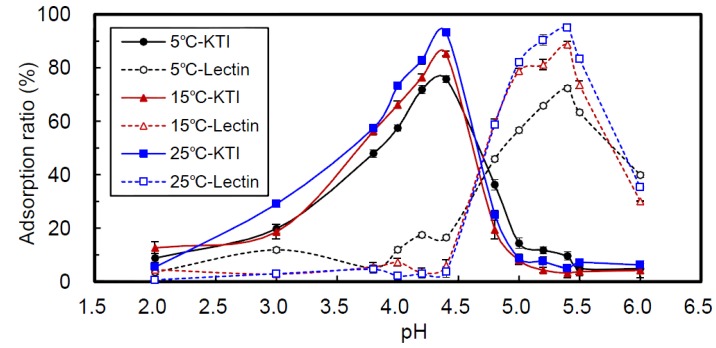
Effects of pH and temperature on the adsorption ratio (%) of chitosan/TPP/genipin bead (CB7) adsorbed to KTI or lectin from soybean whey over 24 h.

The chitosan beads (CBs) were co-crosslinked with TPP/genipin in solutions at pH 5, 7 or 9, and are referred to as CB5, CB7 and CB9, respectively. Figure 2 shows the adsorption ratios of CB7-adsorbed KTI and lectin from soybean whey at 5, 15 and 25 °C for 24 h, over the range of pH 2 to 6. The results reveal that the maximum adsorption ratios of KTI and lectin were at pH 4.4 and 5.4, respectively, regardless of temperature. The pIs of KTI and lectin are pH 4.5 and 5.8, respectively [3,7]. Thus, the maximum adsorption ratios of KTI and lectin were close to their respective pIs.

Feng *et al.* [21] reported that both ovalbumin and lysozyme could be effectively adsorbed by the chitosan/carboxymethylchitosan amphoteric membrane. The lysozyme had a large adsorption capacity at pH 8.0–9.2 due to the positive charge of the lysozyme and the negative charge of the blend membrane. Therefore, the electrostatic interaction between lysozyme and membrane was strong enough to generate effective and significant adsorption. The maximum adsorption capacity of ovalbumin occurred at pH 5.2. Because the ovalbumin is negatively charged and the membrane positively charged, the electrostatic interaction between them was the main force governing adsorption. However, weaker adsorption capacity was still observed between the ovalbumin and the membrane at pH 4.6, the pI of the ovalbumin. Thus, they considered that the carboxyl and hydroxyl groups of the ovalbumin formed hydrogen bonds with the amino and/or hydroxyl groups on the membrane, which was an additional force contributing to adsorption beside the electrostatic interaction.

In this study, the degrees of crosslinking of CB5, CB7 and CB9 were 74.6%, 62.3% and 59.2%, respectively. CBs with different properties, such as ratio of TPP/genipin and crosslinking degree, could be prepared by varying the pH of the solution. In acidic solution, Mi *et al.* [26] demonstrated that chitosan hardly reacts with genipin due to charged amino group of chitosan and they only demonstrated ionic crosslinking with TPP. However, at pH values higher than 6.5, the electrostatic interaction among chitosan and TPP was rare; the crosslink reaction between chitosan and genipin was predominant [26]. Therefore, in pH < 6.5 solution, CBs are amphoteric beads which simultaneously possess positive (–NH^3+^) and negative groups (P_3_O_10_^5−^), contributed from chitosan and TPP respectively. The TPP content of CB7 and CB9 was lower than that of CB5, while the genipin content of CB7 and CB9 was higher than that of CB5.

The main driving force of adsorption of proteins to chitosan-based adsorbents is inferred to be electrostatic attraction, *i.e.*, chitosan contains positively charged groups and proteins contain negatively charged groups [20,21,22]. The system in our study, however, may be different. The adsorption of CBs proceeded at pH < 6.5. In a solution at a pH lower than its pI, the net charge of a protein is positive; thus electrostatic repulsion between proteins and the –NH^3+^ of CBs can occur, thus resulting in a low adsorption ratio. When a protein is in a solution at a pH higher than its pI, the net charge of the protein is negative; electrostatic repulsion between the protein and the P_3_O_10_^5−^ of CBs can occur. This also results in a low adsorption ratio. The best adsorption ratios of CB to KTI and lectin were at pH 4.4 and 5.4, both slightly lower than their pIs (pH 4.5 and 5.8), respectively. This indicates that the main interaction between CBs and proteins could be H-bonding, while electrostatic attraction may be trivial. There are two hydroxyl groups each in the glucopyranose ring of chitosan and in the structure of genipin. These hydroxyl groups can interact with hydroxyl and carboxyl groups of proteins to form H-bonds, improving the adsorption of CBs to proteins. The electrostatic interaction includes attractive and repulsive forces. The electrostatic repulsion may play a more important role than electrostatic attraction in adsorbing CBs to proteins because the adsorption ratios decreased quickly as the pH of solution decreased below or increased above their pIs. Additionally, chitosan (80% degree of deacetylation) includes 20% hydrophobic *N*-acetyl groups. They could interact with hydrophobic groups on protein probably to form hydrophobic interactions and enhance the CBs’ ability to adsorb proteins. However, in general the hydrophobic groups are hidden in the protein’s interior; consequently, the significance of hydrophobic interactions in CBs adsorbing proteins needs more in-depth study. 

**Figure 3:**
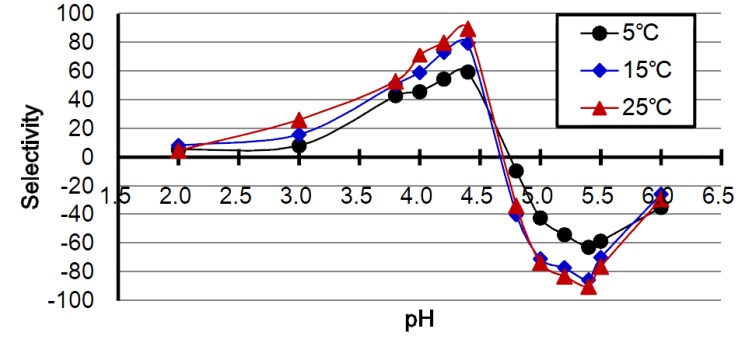
Effects of pH and temperature on the selectivity of chitosan/TPP/genipin bead (CB7) in adsorbing KTI and lectin from soybean whey. The selectivity was defined as adsorption ratio of KTI minus adsorption ratio of lectin at a given pH.

Figure 3 shows the selectivity of CB7 in adsorbing KTI or lectin in soybean whey solution at 5, 15 and 25 °C, over a range of pH 2 to 6. The selectivity was defined as adsorption ratio of KTI minus adsorption ratio of lectin at a given pH. The result indicates a positive peak at pH 4.4 and a negative peak at pH 5.4. This means that the adsorption ratio of KTI at pH 4.4 was much higher than that of lectin, while the adsorption ratio of lectin at pH 5.4 was much higher than that of KTI. Therefore, utilizing the property of pIs, selective adsorption of KTI and lectin with CBs is achieved feasibly through pH modulation.

### 2.2. Effect of Temperature

**Figure 4:**
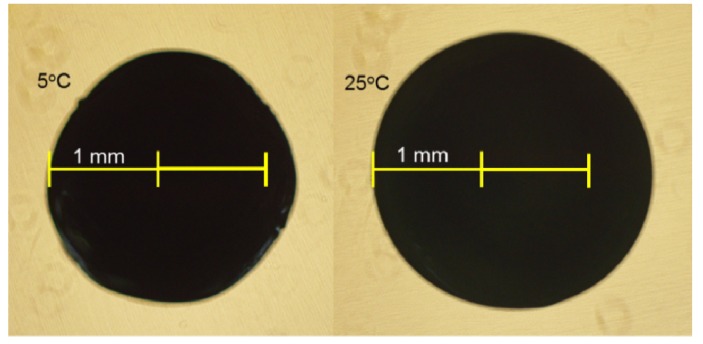
Chitosan/TPP/genipin co-crosslinked beads as visualized by inverted microscope.

Under the same pH, adsorption ratio of CB7 to KTI or lectin at 25 °C was higher than that at 15 and 5 °C (Figure 2), which could be caused by thermodynamic effects. At low temperatures, molecular motions were slow resulting in lower adsorption ratios. Furthermore, Figure 4 shows the size of CB7 in photographs taken through an inverted microscope at 5 and 25 °C. The size and surface of CB7 were larger at 25 °C than at 5 °C. Although the CBs had co-crosslinked with genipin and TPP, its property of thermal expansion and contraction still occurred. This allowed the adsorption ratios to increase as the temperature increased.

### 2.3. Effect of Kinds of CB

**Figure 5:**
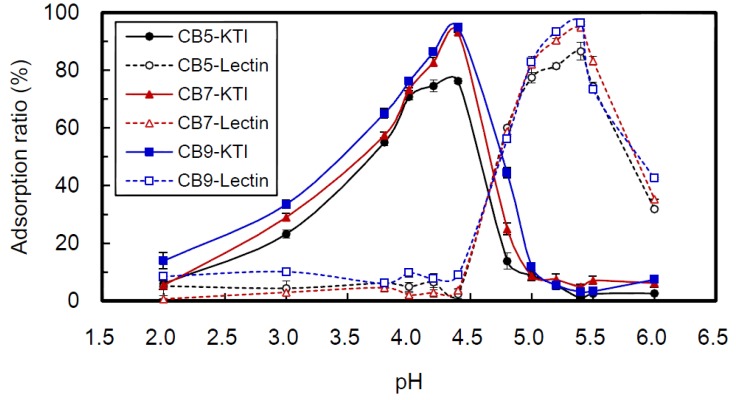
At 25 °C, effects of pH and kinds of beads (CB5, CB7 and CB9) on the adsorption ratio (%) of chitosan/TPP/genipin beads adsorbed to KTI or lectin from soybean whey over 24 h.

Adsorption ratios of CBs to KTI or lectin in soybean whey solution (25 °C) were determined (Figure 5). Over a range of pH 2 to 6 of the soybean whey solution, the trends of adsorption ratios were similar for all three CBs (CB5, CB7 and CB9). The best adsorption ratios for KTI occurred at pH 4.4, and for lectin at pH 5.4. The adsorption ratios of CB5, CB7 and CB9 to KTI at pH 4.4 were 76.3%, 93.2% and 94.8%, and to lectin at pH 5.4 were 86.6%, 94.9% and 96.5%, respectively. The result may relate to crosslinking degree and type of CBs. The crosslinking degree of CB9 was 59.2%, leaving 40.8% of the amino groups free. In comparison, CB5 and CB7 were 74.6% and 62.3% crosslinked, with 25.4% and 37.7% of amino groups free, respectively. The ratios of TPP and genipin crosslinked with chitosan were CB5 > CB7 > CB9 and CB5 < CB7 ≤ CB9, respectively [26]. Interestingly, CB5 had the least amount of hydroxyl groups, while CB7 and CB9 were equivalent. Overall, the adsorption ratio of CBs to protein (KTI or lectin) was the highest for CB9, followed by CB7 then CB5 (Figure 5). Therefore, these results indicate that H-bonding was indeed the main interaction governing adsorption. In addition, in the ranges from pH 4.0 to 4.4 and from 5.0 to 5.4, the slope of the curves was CB5 < CB7 ≤ CB9. This may be due to CB5 having fewer free amino groups resulting in a weaker level of positive charge in the beads. Furthermore, as the pH decreases below the pI, the degree of positive charge of a protein increases Therefore, the electrostatic repulsion between proteins and beads were CB5 < CB7 ≤ CB9 at most pH conditions, which may explain the trend observed for the adsorption ratio (CB5 < CB7 ≤ CB9). These above results again illustrate that the main adsorption force between CBs and KTI or lectin was hydrogen bonding, while electrostatic repulsion suppressed adsorption of CBs to the proteins.

### 2.4. Desorption of CB

**Figure 6:**
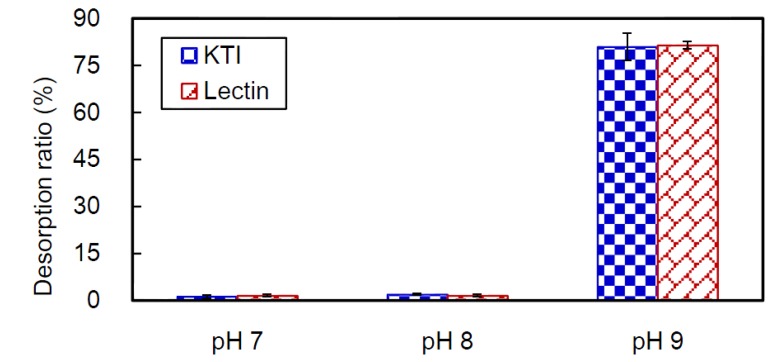
Desorption ratios of KTI and lectin from CB7 at pH 7, 8 and 9 after 24 h at 25 °C. KTI and lectin were initially adsorbed to CB7 under pH 4.4 and 5.4, respectively.

CB7 was adsorbed to KTI or lectin in soybean whey solution (pH 4.4 or 5.4, respectively) for 24 h, at 25 °C. Next, the proteins were desorbed in solutions at pH 7, 8 or 9 for 24 h. Desorption ratios of KTI and lectin from CB7 were determined. As shown in Figure 6, KTI and lectin were hardly desorbed from CB7 at a pH of 7 or 8. However, these proteins easily desorbed from the beads at pH 9, where the desorption ratio of KTI was 80.9%, and that of lectin was 81.4%.

Yang *et al.* [25] reported that the desorption ratios of phytic acid from CB7, at 25 °C for 24 h, were 74.07%, 80.25% and 93.98% at pH 7, 8 and 9, respectively. In higher pH solution, the electrostatic force between the amino groups of chitosan and the phytic acid was weaker due to lower protonation of chitosan. This led to phytic acid more easily desorbing from CB7.

However, KTI and lectin desorb from CB7 by a mechanism different from that of phytic acid. At pH > 7, the amino group of chotosan does not protonize, and can interact with a protein to form a H-bond; this scenario is not conducive to desorption. When pH increases, the TPP becomes more negatively charged, and the net charge level of KTI and lectin is stronger. The low desorption ratios in pH 7 and 8 solutions were perhaps due to the electrostatic repulsions being weaker than the attractive force of the H-bonds forming between proteins and CB7. As the pH increased to 9, the stronger electrostatic repulsion surmounted the attraction of the H-bonding, and caused considerable quantities of proteins to desorb from CB7.

Taken together, our results indicate that CB7 can selectively adsorb KTI (at pH 4.4) and lectin (at pH 5.4) from soybean whey solution. Then, the bulk of KTI and lectin can be desorbed from the beads under solution at pH 9. The recovery ratios of KTI and lectin were up to 76% and 73%, respectively. Thus, our protocol utilizing CB7 is an effective method for the selective isolation of KTI and lectin from soybean whey.

## 3. Experimental Section

### 3.1. Materials

Squid (*Illex argentinus*) pens and soybean (*Glycine max* Merrill) were donated by Shin Dar Bio-Tech. Co., Ltd. (Taoyuan, Taiwan) and Hua Shang Food Enterprise Co., Ltd. (Taoyuan, Taiwan), respectively. The chitosan (degree of deacetylation: 80%, molecular weight: 400 kDa) was prepared from squid pens as described by Tsai *et al.* [27]. Potassium bromide, acetic acid, and iron (III) chloride hexa hydrate were purchased from Merck (Darmstadt, Germany). Sodium tripolyphosphate (TPP), sodium hydroxide, sodium azide, Kunitz trypsin inhibitor, lectin, and sodium acetate anhydrous were purchased from Sigma-Aldrich (St. Louis, MO, USA). Ninhydrin was purchased from Mallinckrodt Baker (Phillipsburg, NJ, USA). Genipin was purchased from Challenge Bioproducts (Taichung, Taiwan).

### 3.2. Preparation of TPP/Genipin Co-Crosslinked Chitosan Beads

The chitosan solution (1%) was applied through a 25 G × 1'' syringe needle mounted by the syringe pump at a controlled flow rate of 0.42 mL/min into 0.01 M TPP/0.01 M genipin solution with pH 5, 7, or 9 (referred to as CB5, CB7 and CB9, respectively) and stored for 24 h to allow co-crosslinking to proceed. After crosslinking, the solidified beads were stirred for two days in ultrapure water (Millipore, Billerica, MA, USA) to remove residual TPP and genipin, and then stored at 4 °C [26].

### 3.3. Determination of Crosslinking Degree of Chitosan Bead

The degree of crosslinking of the chitosan beads was determined by the ninhydrin assay, which can determine the percentage of free amino groups in crosslinked chitosan beads [28]. The ninhydrin solution was prepared as follows: Solution A: 1.05 g citric acid, 10 mL (1 M) NaOH, and 0.04 g SnCl_2_·2H_2_O were mixed, and then the volume was brought up to 25 mL with de-ionized water; Solution B: 1 g ninhydrin was dissolved in 25 mL ethylene glycol monomethyl ether. Solutions A and B were mixed and stirred for 45 min and then stored in a dark bottle. The chitosan beads were lyophilized for 6 h and then weighed. Lyophilized beads (5.0 mg) were placed into a 1.5 mL Eppendorf tube, and then 0.5 mL ninhydrin solution was added. The solution was heated for 20 min at 100 °C and then cooled to room temperature. The mixture was stirred and centrifuged at 4500 rpm for 5 min at 25 °C. Then the absorbance at 570 nm was measured with an ELISA reader (Micro-plate Reader, µQuant-MQX 200, Kcjunior software, Bio-Tec Instruments, Winooski, VT, USA). The concentration of free amino groups was proportional to the absorbance. With d-glucosamine as a standard, a calibration curve was established and the concentration of the free amino groups in the samples was calculated. For each treatment, 3 replicates were performed and the mean values were calculated. The calculation equation for crosslinking degree was as follows:

Crosslinking degree (%) = ((A − B)/A) × 100 (1)

where A is the mole of free amino groups of non-crosslinked chitosan beads, and B is the mole of free amino groups of co-crosslinked chitosan beads.

### 3.4. Preparation of Soybean Whey

The soybean samples were soaked in pure water for 5 h, hulls cleaned, and dried at 45 °C, and then ground into flour. Subsequently the soybean flour was defatted with tenfold weight hexane for 8 h. After parting and drying by fume cupboard, the defatted soybean flour was extracted at room temperature for 2 h with ultrapure water (Millipore, Billerica, MA, USA) adjusted to pH 8 with 2 N NaOH (water:flour = 10:1). It was then centrifuged at 10,400× *g* for 15 min at 20 °C. The supernatant was adjusted to pH 4.5 with 1 N HCl, kept for 2 h at 4 °C, and then subsequently centrifuged at 10,400× *g* for 20 min at 4 °C. The supernatant (pH 4.5) was filtered through No. 1 filter paper (Toyo Roshi Kaisha, Ltd., Tokyo, Japan), adjusted to pH 8 with 2 N NaOH, kept for 1h at room temperature, and then subsequently centrifuged at 12,400× *g* for 15 min at 20 °C. The supernatant was precipitated with ammonium sulphate to 90% saturation, followed by centrifuging at 12,400× *g* for 15 min at 20 °C. The precipitate (soybean whey) was washed with water and dialyzed for 24 h at 4 °C and finally lyophilized (Freeze dry system, Labconco, MO, USA) [3].

### 3.5. Determination of Trypsin Inhibitor and Lectin

The concentration of trypsin inhibitor and lectin was measured with a modified method reported by Castro-Rubio *et al.* [29]. The HPLC system was equipped with L-2130 Pump and L-7455 Detector (Hitachi, Tokyo, Japan). A POROS R2/H perfusion column (100 mm × 2.1 mm I.D., PerSeptive Biosystems, Framingham, MA, USA) was used for the separation of proteins. Mobile phase A consisted of ultrapure water and 0.05% (*v*/*v*) trifluoroacetic acid. Mobile phase B was acetonitrile with 0.05% (*v*/*v*) trifluoroacetic acid. These separations were performed at a flow-rate of 0.5 mL/min (mobile phase A + B) using a solvent gradient. B ratio: 0 min—5%, 8 min—16%, 12.5 min—20%, 13.5 min—24%, 17.5 min—40%, 22.5 min—45%, 26.5 min—50%, 27 min—95%. The injected volume was 20 μL, the operation temperature was 60 °C and UV detection was performed at 254 nm. The standard curves were established via plot of peak area *versus* concentration of trypsin inhibitor or lectin, respectively.

### 3.6. Adsorption and Desorption of KTI and Lectin

After chitosan beads were respectively dipped in blank solutions at different pH values (2–6) for 4 h, the beads were taken out and drained. The 0.1 g CBs was added into 50 mL of pH 2–6 soybean whey solutions. The solutions were stirred at 70 rpm for 24 h at 5, 15 or 25 °C. The concentrations of KTI and lectin in the soybean whey solutions were determined by HPLC before and after adsorption. The calculation equation for adsorption ratio was as follows:

Adsorption ratio (%) = (1 − (*C*1/*C*2)) × 100 (2)

where *C*1 is the amount of KTI or lectin in soybean whey solution after adsorption, and *C*2 is the initial amount of KTI or lectin in soybean whey solution before adsorption.

At 25 °C, the desorption of KTI and lectin from adsorbed CB7 was carried out in 50 mL of pH 7, 8 or 9 solutions, stirred at 70 rpm for 24 h. Finally, the concentrations of KTI and lectin were determined and desorption ratios were calculated. The calculation equation for desorption ratio was as follows:

Desorption ratio (%) = *C*3/*C*4 × 100 (3)

where *C*3 is the desorbed amount of KTI or lectin in solution, and *C*4 is the initial amount of KTI or lectin adsorbed to CB7.

## 4. Conclusions

The maximum adsorption ratios of CBs to KTI and lectin occurred at pH 4.4 and 5.4 respectively, close to their pIs. Fortunately, the selectivity was high at these pHs, *i.e.*, adsorption ratios of other proteins was very low. The adsorption ratios of CBs to KTI and lectin increased with increasing temperature. While CB9 produced the best adsorption ratio compared to CB7 or CB5, the proteins readily desorbed from CB7 (desorption ratio of 80.9% for KTI and 81.4% for lectin). Thus, selective isolation of KTI and lectin from soybean whey can be carried out due to the different pIs of the proteins, and by modulating pH and temperature of the solution. 

The main interaction by which CBs adsorbed KTI and lectin could be H-bonding, while electrostatic attraction was trivial. However, the electrostatic repulsion caused considerable quantities of proteins to desorb from CB7 under pH 9 solution. The degree of crosslinking and the type of CBs apparently affect the forces of adsorption and desorption of beads to adsorbates. Therefore, different characteristics of CBs can be exploited and applied selectively for isolation of different adsorbates. This selective isolation method has potential for applications in large-scale purification of proteins.
